# Complete genome sequence of *Gluconobacter frateurii* ML.ISBL3, an endophytic strain isolated from aerial roots of *Syngonium podophyllum*

**DOI:** 10.1128/mra.01106-23

**Published:** 2024-03-12

**Authors:** M. Lam, K. M. Leung, G. K. K. Lai, F. C. C. Leung, S. D. J. Griffin

**Affiliations:** 1 Shuyuan Molecular Biology Laboratory, The Independent Schools Foundation Academy, Hong Kong SAR, China; University of Strathclyde, Glasgow, United Kingdom

**Keywords:** *Gluconobacter frateurii*, endophytes, plant growth promotion, aerial roots

## Abstract

The endophytic strain *Gluconobacter frateurii* ML.ISBL3 was isolated from aerial roots of *Syngonium podophyllum* in Hong Kong. Its complete genome, established through hybrid assembly, comprises a single chromosome of 3,309,710 bp (56.30% G+C).

## ANNOUNCEMENT

The partial aerobic oxidations of sugars and alcohols to acetic acid by family Acetobacteraceae (1) find use in industrial biotransformations and biosensors (2, 3), although some strains produce valuable exopolysaccharides (4). *Gluconobacter* spp., however, accumulate gluconic acid rather than acetate (5) via membrane-bound pyrroloquinoline-quinone-dependent dehydrogenases (6). Preferring sugar-rich and relatively low-nitrogen environments (1), *Gluconobacter* spp. are commonly associated with plants (7–9), plant-feeding insect guts (10), and the spoilage of soft drinks and juices (11, 12); only rarely are they sources of infection (13, 14).

Here, *G. frateurii* ML.ISBL3 was isolated from aerial roots of *Syngonium podophyllum* in Hong Kong (22.266462N 114.130191E; on 25 June 2021) as a potential symbiont to this successful oligotroph: 20 mm lengths of root tips were surface sterilized using 1% (wt:vol) 8-hydroxyquinoline sulphate before incubation in nitrogen-free Jensen’s broth M710 (15, 16) for 7 days at 32°C. Following subculture to fresh M710 broth and incubation under the same conditions, 100 µL aliquots were spread on M710 agar and Pikovskaya’s agar (17). ML.ISBL3 produces large, clear, mucoid colonies on both media, as well as clear zones on Pikovskaya’s agar. The isolate does not grow well on Luria agar (18), so a single colony taken from M710 agar was passaged to purity on the same medium before streaking onto V8 agar (https://wiki.bugwood.org/V8_agar) for overnight growth before harvesting for DNA extraction (DNeasy PowerSoil Pro Kit, Qiagen GmbH, Hilden, Germany, following manufacturer’s protocol). All agar incubations conducted at 27°C.

Paired-end short-read sequencing libraries were prepared using a NexteraXT DNA Library Preparation Kit (Illumina, Inc., USA) and sequenced via the Illumina MiSeq platform using v3 chemistry (2 × 300 bp). A total of 673,771 raw read pairs were quality filtered and trimmed using TrimGalore! v0.6.7 (https://github.com/FelixKrueger/TrimGalore) (stringency:3; -e:0.2), producing 669,098 read pairs (median length 172 bp) totaling ~237.2 Mbp (SRX21353896). Long-read libraries, prepared from the same extracted DNA using the Rapid Barcoding Kit SQK-RBK004 (without size selection), were sequenced via Oxford Nanopore’s Spot-ON Flow Cell (vR9), MinION sequencer, and MinKNOW v3.1.8 software, with base-calling by Guppy v2.1.3 high-accuracy mode. The final long-read data set of 326,952 reads (N50 10,361) was trimmed by Filtlong v0.2.1 (https://github.com/rrwick/Filtlong) (min_length: 2,000; target_bases: 500 Mbp; length_weight: 10) selecting 81,999 reads (500 Mbp; median length 4,609 bp, N50 7,586) (SRX21353897) to scaffold the assembly. Default parameters were used for all software unless otherwise specified.

Hybrid assembly, overlap removal, and sequence rotation were performed by Unicycler v0.4.8-beta (19) to yield a single circular chromosome of 3,309,710 bp (56.30% G + C; 151× coverage, rotated to begin M9M3E7_GLUTH *dnaA*) judged 100% complete (0% contamination) by CheckM v1.1.6 (20) that was submitted to NCBI PGAP v6.0 (21) for annotation. JSpecies v4.1.1 (22) found ML.ISBL3 closest to *Gluconobacter frateurii* strain NBRC 3264 (BEWN01000000.1) with an average nucleotide identity of 96.91% [ANIb by BLAST +2.2.29 (23)].

PGAP predicts a type 1-levansucrase (24) encoded at locus MKW11_04725, with levanase at MKW11_05395. Similarly, the chemotaxis gene cluster (*che1*) (MKW11_07970–08005) and hopanoid biosynthesis operon *hpnABCDEFG* (MKW11_14220–14260) may support root invasion (25–27), and 1-aminocyclopropane-1-carboxylate deaminase (MKW11_07600) and the *pqqABCDE* operon (MKW11_05315–05295) may promote plant growth (28–30).

## Data Availability

Sequence data for *Gluconobacter frateurii* ML.ISBL3 are available through NCBI under BioProject PRJNA810251, with GenBank and SRA accession numbers CP092689, and SRX22512550 (MinION) and SRX22512549 (MiSeq).

## References

[1] Kersters K, Lisdiyanti P, Komagata K, Swings J. 2006. The family Acetobacteraceae*:* the *genera Acetobacter, Acidomonas, Asaia, Gluconacetobacter, Gluconobacter,* and *Kozakia*. In Dworkin M, Falkow S, Rosenberg E, Schleifer KH, Stackebrandt E (ed), The Prokaryotes. Springer. New York, NY.

[2] Raspor P, Goranovic D. 2008. Biotechnological applications of acetic acid bacteria. Crit Rev Biotechnol 28:101–124. doi:10.1080/07388550802046749 18568850

[3] Svitel J, Tkác J, Vostiar I, Navrátil M, Stefuca V, Bucko M, Gemeiner P. 2006. Gluconobacter in biosensors: applications of whole cells and enzymes isolated from Gluconobacter and Acetobacter to biosensor construction. Biotechnology Letters 28:2003–2010. doi:10.1007/s10529-006-9195-3 17072528

[4] Wünsche J, Schmid J. 2023. Acetobacteraceae as exopolysaccharide producers: current state of knowledge and further perspectives. Front Bioeng Biotechnol 11:1166618. doi:10.3389/fbioe.2023.1166618 37064223 PMC10097950

[5] Asai T, Shoda K. 1958. The taxonomy of Acetobacter and allied oxidative bacteria. J Gen Appl Microbiol 4:289–311. doi:10.2323/jgam.4.289

[6] Yakushi T, Terada Y, Ozaki S, Kataoka N, Akakabe Y, Adachi O, Matsutani M, Matsushita K. 2018. Aldopentoses as new substrates for the membrane-bound, pyrroloquinoline quinone-dependent glycerol (polyol) dehydrogenase of Gluconobacter sp.. Appl Microbiol Biotechnol 102:3159–3171. doi:10.1007/s00253-018-8848-1 29468297

[7] Yukphan P, Charoenyingcharoen P, Malimas S, Muramatsu Y, Nakagawa Y, Tanasupawat S, Yamada Y. 2020. Gluconobacter aidae sp. nov., an acetic acid bacterium isolated from tropical fruits in Thailand. Int J Syst Evol Microbiol 70:4351–4357. doi:10.1099/ijsem.0.004292 32584749

[8] Martínez-Rodríguez J del C, De la Mora-Amutio M, Plascencia-Correa LA, Audelo-Regalado E, Guardado FR, Hernández-Sánchez E, Peña-Ramírez YJ, Escalante A, Beltrán-García MJ, Ogura T. 2014. Cultivable endophytic bacteria from leaf bases of Agave tequilana and their role as plant growth promoters. Braz J Microbiol 45:1333–1339. doi:10.1590/s1517-83822014000400025 25763038 PMC4323307

[9] Berza B, Sekar J, Vaiyapuri P, Pagano MC, Assefa F. 2022. Evaluation of inorganic phosphate solubilizing efficiency and multiple plant growth promoting properties of endophytic bacteria isolated from root nodules Erythrina brucei. BMC Microbiol 22:276. doi:10.1186/s12866-022-02688-7 36401227 PMC9675159

[10] Crotti E, Rizzi A, Chouaia B, Ricci I, Favia G, Alma A, Sacchi L, Bourtzis K, Mandrioli M, Cherif A, Bandi C, Daffonchio D. 2010. Acetic acid bacteria, newly emerging symbionts of insects. Appl Environ Microbiol 76:6963–6970. doi:10.1128/AEM.01336-10 20851977 PMC2976266

[11] Silva MMN, Holanda VL, Pereira KS, Coelho MAZ. 2022. Microbiological contamination profile in soft drinks. Arch Microbiol 204:194. doi:10.1007/s00203-022-02801-4 35217916

[12] Lott AF, Carr JG. 1964. Characteristics of an organism causing spoilage in an orange juice beverage. J Appl Microbiol 27:379–384. doi:10.1111/j.1365-2672.1964.tb05045.x

[13] Alauzet C, Teyssier C, Jumas-Bilak E, Gouby A, Chiron R, Rabaud C, Counil F, Lozniewski A, Marchandin H. 2010. Gluconobacter as well as Asaia species, newly emerging opportunistic human pathogens among acetic acid bacteria. J Clin Microbiol 48:3935–3942. doi:10.1128/JCM.00767-10 20826638 PMC3020812

[14] Olenski̇ M, Brenton L, Taylor L, Papaluca T, Crowe A. 2020. A case of Gluconacetobacter liquefaciens bacteremia associated with sugarcane juice ingestion. JMID 10:62–67. doi:10.5799/jmid.700538

[15] Jensen HL. 1942. Nitrogen fixation in leguminous plants. II. is symbiotic nitrogen fixation influenced by Azotobacter? Proceedings of the Linnean Society of New South Wales, Vol. 67, p 205–212. https://www.biodiversitylibrary.org/page/34945797

[16] Ranganayaki S, Mohan C. 1981. Effect of sodium molybdate on microbial fixation of nitrogen. Z Allg Mikrobiol 21:607–610. https://pubmed.ncbi.nlm.nih.gov/7331378/. 7331378

[17] Pikovskaya RI. 1948. Mobilisation of phosphorus in soil in connection with vital activity of some microbial species. Microbiologiya 17:362–370.

[18] MacWilliams MP, Liao MK, American Society for Microbiology. 2006. Luria broth (LB) and Luria agar (LA) media and their uses protocol. ASM MicrobeLibrary. https://asm.org/Protocols/Luria-Broth-LB-and-Luria-Agar-LA-Media-and-Their-U.

[19] Wick RR, Judd LM, Gorrie CL, Holt KE. 2017. Unicycler: resolving bacterial genome assemblies from short and long sequencing reads. PLoS Comput Biol 13:e1005595. doi:10.1371/journal.pcbi.1005595 28594827 PMC5481147

[20] Parks DH, Imelfort M, Skennerton CT, Hugenholtz P, Tyson GW. 2015. CheckM: assessing the quality of microbial genomes recovered from isolates, single cells, and metagenomes. Genome Res 25:1043–1055. doi:10.1101/gr.186072.114 25977477 PMC4484387

[21] Haft DH, DiCuccio M, Badretdin A, Brover V, Chetvernin V, O’Neill K, Li W, Chitsaz F, Derbyshire MK, Gonzales NR, Gwadz M, Lu F, Marchler GH, Song JS, Thanki N, Yamashita RA, Zheng C, Thibaud-Nissen F, Geer LY, Marchler-Bauer A, Pruitt KD. 2018. RefSeq: an update on prokaryotic genome annotation and curation. Nucleic Acids Res 46:D851–D860. doi:10.1093/nar/gkx1068 29112715 PMC5753331

[22] Richter M, Rosselló-Móra R, Oliver Glöckner F, Peplies J. 2016. JSpeciesWS: a web server for prokaryotic species circumscription based on pairwise genome comparison. Bioinformatics 32:929–931. doi:10.1093/bioinformatics/btv681 26576653 PMC5939971

[23] Camacho C, Coulouris G, Avagyan V, Ma N, Papadopoulos J, Bealer K, Madden TL. 2009. BLAST+: architecture and applications. BMC Bioinformatics 10:421. doi:10.1186/1471-2105-10-421 20003500 PMC2803857

[24] Jakob F, Quintero Y, Musacchio A, Estrada‐de los Santos P, Hernández L, Vogel RF. 2019. Acetic acid bacteria encode two levansucrase types of different ecological relationship. Environmental Microbiology 21:4151–4165. doi:10.1111/1462-2920.14768 31374141

[25] Guo T, Zhou Y, Xie Z, Meng F. 2023. The two chemotaxis gene clusters of Ensifer alkalisoli YIC4027T, a symbiont of Sesbania cannabina play different roles in chemotaxis and competitive nodulation. Agronomy 13:570. doi:10.3390/agronomy13020570

[26] Pan JJ, Solbiati JO, Ramamoorthy G, Hillerich BS, Seidel RD, Cronan JE, Almo SC, Poulter CD. 2015. Biosynthesis of squalene from farnesyl diphosphate in bacteria: three steps catalyzed by three enzymes. ACS Cent Sci 1:77–82. doi:10.1021/acscentsci.5b00115 26258173 PMC4527182

[27] Belin BJ, Busset N, Giraud E, Molinaro A, Silipo A, Newman DK. 2018. Hopanoid lipids: from membranes to plant-bacteria interactions. Nat Rev Microbiol 16:304–315. doi:10.1038/nrmicro.2017.173 29456243 PMC6087623

[28] Choi O, Kim J, Kim JG, Jeong Y, Moon JS, Park CS, Hwang I. 2008. Pyrroloquinoline quinone is a plant growth promotion factor produced by Pseudomonas fluorescens B16. Plant Physiol 146:657–668. doi:10.1104/pp.107.112748 18055583 PMC2245851

[29] Orozco-Mosqueda MDC, Glick BR, Santoyo G. 2020. ACC deaminase in plant growth- promoting bacteria (PGPB): an efficient mechanism to counter salt stress in crops. Microbiol Res 235:126439. doi:10.1016/j.micres.2020.126439 32097862

[30] Bhanja E, Das R, Begum Y, Mondal SK. 2021. Study of pyrroloquinoline quinine from phosphate-solubilizing microbes responsible for plant growth: in silico approach. Front Agron 3:667339. doi:10.3389/fagro.2021.667339

